# How Hospital Income May Be Affected

**Published:** 1912-03-30

**Authors:** 


### HOW HOSPITAL INCOME MAY BE AFFECTED.

### Workmen, the Voluntary Hospitals, and the Insurance Act.

### THE CORNISH MINERS' VIEW.

We have received the following letter signed by G. Phillips Paige, Hon. Secretary to the West Cornwall Miners' and Women's Hospital, Redruth, which has been addressed to the Chairman of the Insurance Commissioners:—

"The Committee of the above hospital have had under their consideration the probable effect of the Insurance Act, 1911, upon this hospital, which is mainly supported by voluntary subscriptions, over £300 (*i.e.* one-sixth of the total income) being contributed by miners employed in the neighbouring mines.

"The Committee are of opinion that the compulsory payments by the employers under the Act will result in a decrease of the contributions by them, and the compulsory payments by the employed in the total loss of the £300 from the miners, who cannot be expected to pay voluntarily towards the hospital when they are paying compulsorily for complete medical attendance. More than an average number of serious accidents are brought to the hospital for attention which cannot be dealt with in the miners' homes with any chance of success.

"The great majority of insured persons admitted to the hospital have dependants and therefore will not be within the scope of section 12 (*c*) of the Act, and it is very unlikely that any approved Society or Insurance Committee will be able or willing to grant such subscriptions to the hospital under section 21 as will compensate for the loss of income.

"Under these circumstances the Committee strongly urge that the Act should be amended so as to provide for payments to hospitals in respect of insured persons who are inmates thereof in every case, but not exceeding the cost which the hospital may incur in each such case."

### THE BIRKENHEAD EMPLOYEES' COUNCIL'S OPINION.

It will be remembered that we published recently a resolution passed at a special meeting of this council in regard to the National Insurance Act and its bearings upon the voluntary hospitals. That resolution was as follows:—

"That this meeting, which represents a very large proportion of the working men of Birkenhead, who contributed last year £1,440 4s. 5d. towards the total income of £4,416 11s. 11d. received by the hospital, is firmly convinced that when the National Insurance Act comes into operation there will be a very serious falling off in the workmen's contributions, and earnestly urges that Parliament will see its way to pass an amending Act, providing that out of the insurance fund such grant may be made to voluntary hospitals as they may need for their due support on their present basis."

We shall be glad to have further communications on this subject from individual hospitals and from organisations of workmen who support these institutions.

### THE LORD MAYOR OF SHEFFIELD'S VIEW.

The Lord Mayor of Sheffield recently made an interesting speech dealing with the probable effect of the Insurance Act upon the funds of voluntary hospitals. He was afraid, said his lordship, the lot of hospitals dependent on individual subscriptions would be more difficult in the future than in the past. Without in any way wishing to trench upon political subjects, he said it must be in the minds of the boards of all hospitals that an Act had been passed which called upon the whole of the working-class community to set aside a portion of their income for sickness in a larger measure than they were previously called upon to do. And it was only reasonable to suppose that when a weekly deduction was made from wages for sickness benefit, the voluntary deduction that the working-classes had very generously made for hospital maintenance would somewhat substantially suffer. Those who had supported the hospitals by voluntary subscriptions were also in the position that they were called upon to share more heavily in the burdens for sickness insurance in the future than in the past, and he was afraid there might be a spirit of economy in the minds of many who had previously given, because of the burdens of the Insurance Act. He trusted, therefore, that the generosity of the English people would be equal to sustaining the voluntary hospital in addition to bearing those burdens of taxation placed upon them by the new Act. The hospitals would be even more necessary to the community in future, because the result of the investigations into the health of the community under the Insurance Act would, undoubtedly, be to discover more cases requiring institutional treatment, and consequently the probability was that there would be a greater demand than ever for the accommodation of hospitals. He knew that under the new Act the new health committees were entitled to subscribe to hospitals, but whilst he was not going to suppose for one moment that those health committees would not desire to be as generous to hospitals as the working-class had been, he was quite aware that in starting any new scheme there must be a sense of doubt as to whether the amount received would be equal to the expenditure, and a sense of prudence and care in giving away moneys. The health committees were bound to take a somewhat careful view of the situation until they knew what surplus they would have, and, therefore, it would be an anxious time for the hospitals when the new Act came into force. He appealed for the more generous support of hospitals at a time when the friends of the hospitals most needed to rally round them, and trusted that he would not appeal in vain.

The Lord Mayor might have added to his interesting remarks the fact that the funds available for subscription to hospitals under the National Insurance Act, as it stands, must be so small as to be inappreciable so far as any benefit to the voluntary hospitals is concerned. The Act makes no adequate provision for hospital benefit and practically no provision at all.

